# Evaluation of serum and tissue levels of VAP-1 in colorectal cancer

**DOI:** 10.1186/s12885-016-2183-7

**Published:** 2016-02-24

**Authors:** Stephen T. Ward, Christopher J. Weston, Emma L. Shepherd, Rahul Hejmadi, Tariq Ismail, David H. Adams

**Affiliations:** Centre for Liver Research & NIHR Birmingham Biomedical Research Unit, Level 5 Institute for Biomedical Research, University of Birmingham, Vincent Drive, Birmingham, B15 2TT UK; Department of Colorectal Surgery, Queen Elizabeth Hospital, Mindelsohn Way, Edgbaston, Birmingham, B15 2GW UK

**Keywords:** VAP-1, AOC3, Colorectal Cancer, Tumor Markers

## Abstract

**Background:**

The endothelial adhesion molecule, vascular adhesion protein-1 (VAP-1, *AOC3*) promotes lymphocyte recruitment to tumours, although the contribution that VAP-1 makes to lymphocyte recruitment in human colorectal cancer (CRC) is unknown. VAP-1 exists in circulating soluble form (sVAP-1). A previous study demonstrated elevated sVAP-1 levels in CRC patients. The aim of this study was to confirm this finding and study the differences in tissue VAP-1 expression between CRC and healthy tissues.

**Methods:**

sVAP-1 levels were measured in the serum of 31 patients with CRC and 31 age- and sex-matched controls. Tissue VAP-1 levels were measured by immunohistochemistry, quantitative real-time PCR and Western blotting.

**Results:**

The mean sVAP-1 level ± SD was significantly lower in the CRC group compared with the control group (399 ± 138 ng/ml versus 510 ± 142 ng/ml, *P* = 0.003). Tissue VAP-1 protein and mRNA levels were significantly lower in CRC compared with normal colon tissue. VAP-1 immunostaining was practically absent from CRC.

**Conclusions:**

VAP-1 is downregulated in human CRC and although the molecular basis of this down regulation is not yet known, we suggest it may be part of a mechanism used by the tumour to prevent the recruitment of anti-tumour immune cells. Our data contradicts the findings of others with regard sVAP-1 levels in patients with CRC. Possible reasons for this are discussed.

## Background

Colorectal cancer (CRC) is the third most common cause of cancer-related deaths in the UK and United States [1, 2]. Lymphocyte infiltration in human CRC is associated with favourable outcome, both in terms of overall survival and recurrence-free survival [3]. More recent analyses have shown that assessment of CD8+ and memory CD45RO+ lymphocyte infiltration rivals the prognostic ability of the standard TNM staging system [4].

Lymphocytes are recruited to tissues from blood via binding to vessels in the target tissue mediated by interactions between specific receptors on the lymphocyte surface and ligands on the endothelium. These interactions involve several families of adhesion molecules including integrins and immunoglobulin superfamily members and selectins and carbohydrate-bearing ligands. The atypical endothelial adhesion molecule vascular adhesion protein-1 (VAP-1, *AOC3*) has been reported to be increased in inflamed mucosal vessels in the gut where it is believed to promote the recruitment of leucocytes [5].

VAP-1 mediates adhesion via heavily sialidated side chains but it also functions as a cell surface expressed ectoenzyme—a primary amine oxidase. As such it deaminates primary amines to form the corresponding aldehyde, ammonia and hydrogen peroxide, although the physiological substrates are not well defined. VAP-1 was originally characterised by a monoclonal antibody (mAb 1B2) that was capable of inhibiting lymphocyte adhesion to high endothelial venules (HEV) and immobilised purified VAP-1 protein [6] but it subsequently transpired that the enzymatic activity of VAP-1 is also involved in its ability to promote transendothelial migration.

In addition to its ability to promote recruitment to the gut via its expression on mucosal vessels, VAP-1 has also been implicated in the recruitment of lymphocytes to several human tumours including head and neck cancer and hepatocellular carcinoma where VAP-1 expressed on tumour endothelium was shown to support lymphocyte binding [7, 8]. In murine models of melanoma and lymphoma, tumours were smaller in VAP-1 knock-out mice compared to wild-type littermates [9] as a consequence of reduced myeloid suppressor cell recruitment. Both MDSC (myeloid-derived suppressor cells) and regulatory T cells are recruited to CRC where they inhibit the anti-tumour immune response, thereby promoting tumour progression. The contribution of VAP-1 to the recruitment of lymphocytes to CRC is unknown. Immunohistochemical studies have demonstrated weak expression of VAP-1 in Peyer’s patches and lamina proprial vessels of the gut with increased staining in inflammatory bowel disease. The staining is typically not as intense as that seen for lymph node and tonsillar HEV, or that observed in other tissues, most notably the hepatic sinusoid [8].

VAP-1 also exists in soluble form, probably generated through cleavage of the membrane-bound protein, and can be found in the sera of healthy human subjects. In contrast to other soluble adhesion molecules, soluble VAP-1 (sVAP-1) levels are not consistently raised in association with inflammatory diseases but rather associated with particular conditions including inflammatory liver disease and diabetes. Increased levels have been reported in one study in CRC compared to controls with reduced levels associated with progressive disease [10], while levels in patients with hepatocellular carcinoma are not significantly raised above controls [11]. A large study has shown an association between sVAP-1 levels and all-cause mortality in CRC, although this was only after adjustment for multilevel confounding variables [12].

The aim of this study was to confirm the reported elevated levels of sVAP-1 in patients with CRC compared with controls and determine if differences observed in sVAP-1 levels are reflected by changes in tissue VAP-1 expression. As the infiltration of specific lymphocyte subsets in CRC has a profound effect on prognosis, alterations in the expression pattern of VAP-1 may provide a means of cancer-specific lymphocyte recruitment.

## Methods

### Patients and samples

Stored serum samples from a historical cohort of adults referred to the colorectal clinic at the University Hospital Birmingham NHS (National Health Service) Foundation Trust, Birmingham, UK were used for measurement of sVAP-1 levels. Patients had been recruited into a previous study evaluating serum tumour markers for CRC, the details of which have been reported in full elsewhere [13, 14]. Patients were eligible to be recruited if they were aged 18 or over and had been referred to the colorectal clinic for evaluation and investigation of lower gastrointestinal symptoms. Recruited patients had a serum sample taken and the results of examination, investigations and the subsequent diagnosis were recorded. Patients had given consent for serum samples to be used in the future for the purpose of evaluation of diagnostic tests and new potential biomarkers. Of 962 patients who had given consent for future use of stored serum samples, 31 patients had proven CRC (‘cases’). Serum from 31 age-, sex-, and ethnic group-matched patients from the remaining 931 patients who were proven to not have CRC formed the control group. Matching was performed by calculation of the general dissimilarity coefficient of Gower, using ‘R’ (R foundation for Statistical Computing, Austria) and the e1071 package [15].

Tissue samples were obtained from patients undergoing resection for CRC having given informed consent at the University Hospital Birmingham NHS Foundation Trust. Fresh pieces of tumour tissue and strips of colonic tissue, a minimal distance of 10 cm from the tumour, were taken with the help of an experienced pathologist.

### Ethics statement

Serum samples were collected from a previous study evaluating CRC tumour markers. Full ethical approval had been obtained from North Birmingham Research Ethics Committee in October 2004 (Ref: 04/Q2704/29) covering use of the samples for this study. Patients had given formal written consent for collection and storage of the serum samples. Ethical approval was obtained for the collection of tissue samples from the Local Research & Ethics Committee (LREC South Birmingham, reference 2003/242, renewed 2012). All patients who donated tissue samples had given formal written consent.

### Enzyme immunoassay for sVAP-1

Serum sVAP-1 was measured by time-resolved immunofluorometric assay at BioTie Therapies Corp., Finland. A biotin-conjugated monoclonal anti-human VAP-1 antibody was adsorbed onto a microtiter plate. Detection of bound serum VAP-1 was performed using a different europium-conjugated anti-human VAP-1 antibody. Time-resolved fluorescence was measured using a fluorometer (Victor^2^Multilabel Counter, PerkinElmer) at 615 nm. Serum VAP-1 concentration was quantified on the basis of a reference sample of highly purified human serum VAP-1 (Biovian Ltd).

### Immunohistochemistry and immunofluorescence

Formalin-fixed and paraffin-embedded tissues were deparaffinised and rehydrated by passing the sections through fresh solutions of Clearene (Leica GmBH, Germany) and graduated alcohols. Sections for immunohistochemistry were incubated with 0.3 % hydrogen peroxide solution in methanol and all sections underwent antigen retrieval by microwaving in pre-heated EDTA buffer (0.37 g EDTA in 1 l distilled water, pH 8.0, 0.05 % Tween 20) for 15 min. Sections were blocked with 10 % casein solution for 30 min and then incubated with either anti-AOC3 antibody (Rabbit polyclonal, HPA000980, Sigma-Aldrich Ltd, UK) at 1 in 100 dilution or an isotype control antibody at equal concentration (Dako Ltd, UK) for 1 h at room temperature. The anti-AOC3 antibody had been developed and validated by the Human Protein Atlas [16]. Tissue sections for immunofluorescence were incubated with primary antibodies against both AOC3 and the endothelial cell marker CD31 (Mouse IgG1, Clone 9G11, R&D Systems, UK). The sections were then washed twice in TBS containing 0.1 % Tween-20 and incubated with an HRP-conjugated development system (Vector ImmPress, Vector Labs) or fluorescent secondary antibody (Alexa-Fluor 546 goat anti-rabbit IgG and Alexa-Fluor 633 goat anti-mouse IgG1, Life Technologies, UK) both at 1 in 500 dilution for 20 min at room temperature. Following a final wash with TBS/0.1 % Tween-20, immunohistochemistry sections were visualised using Novared (Vector Labs Inc, USA) and counter-stained with Meyer’s haematoxylin (Dako Ltd, UK) before mounting in DPX (Leica GmBH, Germany). Immunofluorescent sections were counter-stained with DAPI and mounted with Mowiol (Sigma-Aldrich Ltd, UK). Immunocytochemistry was performed on cultured human colonic endothelial cells, below passage 4, using anti-AOC3 and anti-CD31 antibodies as described above for tissue immunofluorescence. Tissue expression was visualised using a Leica DM6000 (chromogenic) microscope or an LSM510 confocal microscope (Zeiss GmBH, Germany) and the manufacturer’s software.

### Quantitative real-time PCR

RNA was extracted from snap-frozen tissue samples using the Qiagen RNeasy minikit as per manufacturer’s instructions with on-column DNA digestion. The RNA concentration was adjusted to 100 μg/ml following assessment of RNA quantity and quality by UV absorbance at 260/280 nm (Nanophotometer, Implen GmBH, Germany). RNA was reverse transcribed from 3 μg RNA using the iScript kit (Biorad Inc, USA) and quantitative real-time PCR was performed using the LightCycler 480 (Roche, UK). Primers and probes were designed using the RealTime Ready software (Roche) for VAP-1 and two housekeeping gene expression assays: GUSB (glucuronidase, beta) and IPO8 (importin-8). The GUSB assay was obtained from Roche (Universal ProbeLibrary Reference Gene Assay). Other probes were obtained from Roche and reverse-phase purified primers were manufactured by Altabioscience (Birmingham, UK). The following probes and intron-spanning primer sequences were used:

VAP-1 mRNA levels were expressed relative to GUSB and IPO8 mRNA as calculated by the Roche E-method [17]. Amplification conditions were: Pre-incubation at 95 **°**C for 10 min followed by 60 cycles of denaturation at 95 **°**C for 10 s, annealing at 55 **°**C for 30 s and extension at 72 **°**C for 1 s. Analysis was performed on 8 samples of matched colon and CRC. Control reactions containing no reverse transcriptase or no RNA were negative. Differences in relative gene expression between colon and matched CRC samples were tested for statistical significance using a two-tailed unpaired *t*-test.

### Western blotting

Protein was extracted from snap-frozen tissue by incubation in ice-cold lysis buffer (CellLytic MT, 20 μl/mg tissue, Sigma-Aldrich Ltd, UK) containing a proteinase inhibitor cocktail (Sigma-Aldrich Ltd, UK) and 5 U/ml DNase I (Roche, UK). The tissue was mechanically dissociated in the buffer solution using the GentleMACS tissue dissociator (Miltenyi Biotec Ltd, UK). Samples were pelleted and the supernatant collected. Protein concentrations were determined against a bovine serum albumin standard using a bicinchoninic acid assay and sample protein concentrations adjusted to 2 mg/ml by dilution in cell lysis buffer.

40 μg protein per sample was incubated at 98 °C for 1 min in SDS-PAGE sample buffer containing 10 mM beta-mercapto-ethanol, resolved on a 12 % SDS-PAGE gel and then transferred to a nitrocellulose membrane. Membranes were blocked in 5 % non-fat milk dissolved in PBS containing 0.1 % Tween-20 for 1 h at room temperature. Membranes were subsequently incubated overnight at 4 °C with either rabbit anti-human VAP-1 (Sigma-Aldrich Ltd, UK) at1 in 1000 dilution, anti-goat GUSB (R&D Systems Ltd, UK) at1 in 200 dilution or anti-mouse GAPDH (Sigma-Aldrich Ltd, UK) at1 in 10,000 dilution followed by a 1 h room temperature incubation with HRP-conjugated anti-rabbit/goat/mouse antibodies (Dako Ltd, UK; 1 in 2500). Protein bands were detected with the PicoWest ECL system (Pierce, USA). Protein band densities were calculated using ImageJ v1.46r (NIH, USA) and differences in band densities between colon and matched CRC samples were tested for statistical significance using a two-tailed unpaired *t*-test.

### Flow cytometry

Fresh tissue was washed in RPMI containing penicillin (10,000 U/ml), streptomycin (10,000 μg/ml), L-glutamine (29.2 mg/ml), gentamicin (5 mg/ml) and amphotericin (125 μg/ml, Life Technologies Ltd, UK). The tissues were cut into small pieces and digested in a solution containing collagenase (C2-22, VWR International Ltd, UK) for 1 h at 37 °C. The mixture was then passed through a fine mesh to remove debris. An immunomagnetic depletion of EpCAM-expressing cells was performed using EpCAM microbeads and MACS columns (Miltenyi Biotec Ltd, UK) as this is a common cell contaminant in endothelial cell cultures. Positive immunoselection of endothelial cells was then performed using CD31 microbeads and cells were resuspended in endothelial basal medium (Lonza Ltd, UK) supplemented with the aforementioned antibiotics. Cultured endothelial cells, below passage four, from both human colon and CRC were labelled withanti-CD31 (Life Technologies Ltd, UK) and anti-VAP-1 (Clone: TK8-14, Conjugated in our laboratory to APC using the Lynx system; Serotec, UK). HEK293 cells which had been transfected with a GFP-VAP1 construct were used as a positive control for the VAP-1 antibody (as described in Weston et al, JNT 2013 [18]).

## Results

### Serum sVAP-1 levels are lower in patients with CRC compared with controls

**Fig. 1:**
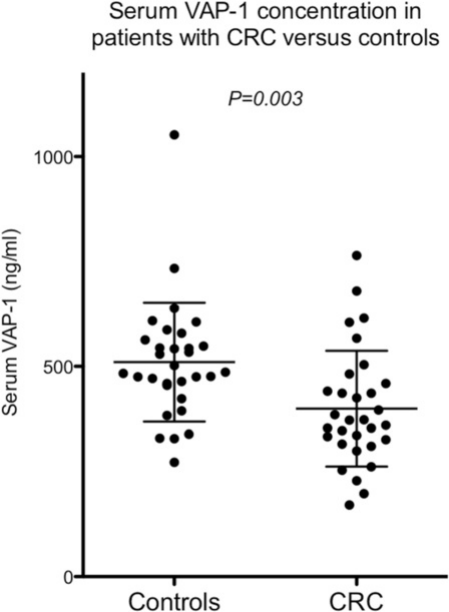
Serum VAP-1 (sVAP-1) levels measured in patients with CRC and controls. The bars indicate the mean ± one standard deviation. Differences between groups were tested for statistical significance using a two-tailed unpaired *t*-test

sVAP-1 levels were measured in the serum of 31 patients with proven CRC and 31 age-, sex- and ethnic group-matched control patients who had been investigated for lower gastrointestinal symptoms and proven not to have CRC. The distribution of sVAP-1 levels was normally distributed and therefore differences in the group means was tested for statistical significance using a two-tailed unpaired *t*-test. The mean sVAP-1 level ± one standard deviation was 399 ± 138 ng/ml in the CRC group compared with 510 ± 142 ng/ml in the control group (*P* = 0.003, Fig. 1).

### Expression of tissue VAP-1 protein and mRNA

**Fig. 2:**
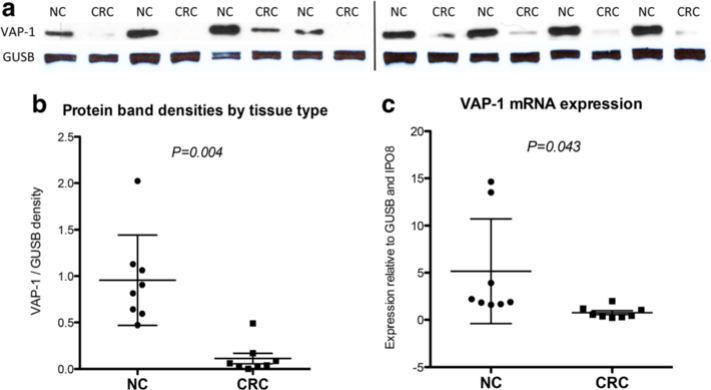
**a** VAP-1 protein expression by Western blot for 8 normal colon (NC) and matched CRC samples. The vertical black line indicates separate gels. **b** Protein band densitometry of VAP-1/GUSB shows a statistically significant reduction in VAP-1 protein expression in CRC tissue compared with matched colon. **c** VAP-1 gene expression by 8 normal colon and CRC samples shows a statistically significant reduction in VAP-1 mRNA expression in CRC tissues compared with matched colon, relative to GUSB and IPO8 expression. The bars indicate the mean ± one standard deviation. Differences between groups were tested for statistical significance using a two-tailed unpaired *t*-test

Western blotting was used to analyse VAP-1 protein expression in tissue lysates generated from tissue taken from patients with CRC or matched normal controls. Levels of VAP-1 protein were consistently lower in CRC tissue when compared to normal tissue in 8 matched samples (Fig. 2a,b). Densitometric analysis of VAP-1 protein bands relative to GUSB bands showed that this difference was highly statistically significant. Equal protein loading was demonstrated by similar GUSB protein band densities for each lane.

Quantitative real-time PCR revealed lower VAP-1 gene (*AOC3*) expression in CRC compared with matched colon for 7 out of 8 samples (Fig. 2c), consistent with the protein expression data. A panel of 11 different housekeeping genes were compared on 8 matched samples analysed using GeNORM, Normfinder and Bestkeeper software to determine the best housekeeping genes with the least variation between tumour and normal colon [Unpublished data]. *AOC3* expression was therefore calculated relative to *GUSB* and *IPO8*, both of which displayed stable expression between samples.

### Immunohistochemistry

**Fig. 3:**
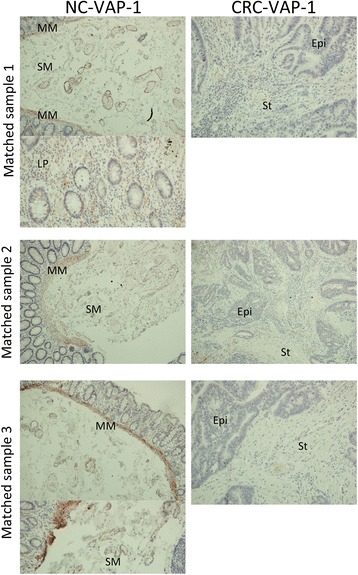
Paraffin-embedded sections from 3 matched normal colon (NC) and CRC samples demonstrating positive VAP-1 staining in the lamina propria and submucosa of the colon with only weak staining in CRC stroma. Magnification x200. VAP-1 staining is brown-red, haematoxylin staining is blue. MM = muscularis mucosae; SM = submucosal; LP = lamina propria; St = tumour stroma; Epi = tumour epithelium

**Fig. 4:**
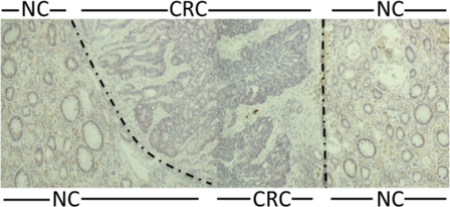
VAP-1 expression in flanking normal colon (NC) tissue abruptly disappears at the border with CRC tissue. Dot-dash line marks the border between NC and CRC. Magnification x200. VAP-1 staining is brown-red, haematoxylin staining is blue

**Fig. 5:**
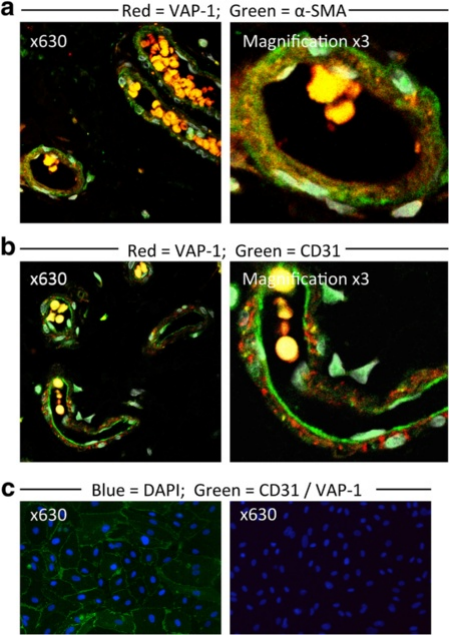
Dual-colour immunofluorescent staining of sections of normal colon tissue and cultured colonic endothelial cells. Magnification x630. **a** VAP-1 staining is present in SMA+ cells of vessels in sections of colonic mucosa. **b** VAP-1 staining does not co-localise with CD31+ endothelial staining of vessels in sections of colonic mucosa. Right-most images are digitally magnified x3. Yellow staining represents autofluorescence from erythrocytes. **c** Positive CD31 staining (left) and absent VAP-1 staining (right) of cultured colonic endothelial cells

VAP-1 tissue expression was determined using immunohistochemical staining of sections from 10 colon and matched CRC samples of different disease stages, all were moderate grade adenocarcinomas. In normal colon VAP-1 was detected in the muscularis mucosae and in vessels in the lamina propria and submucosa (Fig. 3). In contradistinction, VAP-1 staining was practically absent from CRC, apart from occasional weak cytoplasmic staining of the tumour epithelium. A section of CRC with adjoining colon on both sides revealed VAP-1 expression within the colon with an abrupt disappearance at the border between the colon and cancer tissue (Fig. 4). High magnification microscopy of colon tissue suggested that most of the VAP-1 staining detected in vessels was detected in cells surrounding the endothelium, such as pericytes and smooth muscle cells. This was confirmed by dual-colour immunofluorescence, demonstrating localisation of VAP-1 to α-SMA-positive cells rather than CD31-positive cells (Fig. 5a,b). Cultured endothelial cells from human colon expressed cell-surface CD31 but no VAP-1 neither on the cell surface nor in the cytoplasm (Fig. 5c).

## Discussion

We have shown that circulating sVAP-1 levels are significantly lower in patients with CRC compared with controls. This surprising finding directly contradicts a previous study reporting elevated serum sVAP-1 levels in patients with CRC compared to healthy control subjects [10]. In this earlier study, the highest sVAP-1 levels were found in early stage disease while lower levels predicted lymphatic and hepatic metastasis. Serum sVAP-1 levels were generally lower than in our study and this may represent different methods of measurement. In their study, a commercially available ELISA kit was used (Bender MedSystems GmbH, Austria) whereas in our study a well validated, sensitive immunofluorometric assay was used (Biotie Therapies Corp, Finland). In gastric cancer serum sVAP-1 levels measured using the commercial kit are also reportedly elevated compared with controls, with the level declining as the disease progresses [19]. In all studies, the control group has been matched with the case group for age and sex which suggests the method of measurement as a source for the disparity in the results. Another possibility relates to the different study populations: Japanese for the two studies reporting elevated sVAP-1 levels in cancer patients compared to a largely caucasian population (>90 %) in our study. We used a control group of patients referred to the colorectal clinic for various lower gastrointestinal symptoms as detailed previously [14]. This control group is therefore different to the healthy controls described in the other studies [10, 19], but more relevant as a comparator group with CRC patients. Increased levels of sVAP-1 have been reported in inflammatory diseases such as ectopic eczema [20], liver cirrhosis [11] and multiple sclerosis [21]. There is notably no increase in sVAP-1 levels in inflammatory bowel disease compared to healthy controls [22], despite increased tissue levels of VAP-1. A study using the Biotie immunofluorometric assay reported an association between sVAP-1 and all-cause mortality in CRC [12]. This study did not compare sVAP-1 levels between patients with CRC and control patients without CRC. There was also no such association by univariate analysis, only when adjusted for multilevel confounding variables.

Having detected lower levels of sVAP-1 in CRC we wanted to see if this reflected reduced tissue expression. There are very few previous studies of VAP-1 tissue expression by CRC. A recent paper reports increased VAP-1 in patients with CRC but the validity of the observations was reduced by staining in the negative controls and the finding of VAP-1 in the lumen of CRC tissue [12]. The antibody used in this study (Santa Cruz Biotechnology Inc, USA) differs from the one used in our study and could provide one explanation for observed differences in positive VAP-1 staining. We were scrupulous in our choice of controls and compared CRC with normal colon tissue where tissue staining has been reported before. We have also compared the positive immunostaining of the HPA000980 antibody in the liver with other well-validated anti-VAP-1 antibodies such as TK8-14 and similar staining was observed (unpublished observations). This allowed us to be sure that the staining we observed was specific for the tumours. Consistent with the reduced soluble levels of VAP-1 in CRC, we detected a marked reduction in VAP-1 expression as determined by gene expression analysis, protein immunoblotting and immunohistochemistry by CRC compared with normal colon tissue. Indeed there was minimal detection of VAP-1 in CRC. In tissue stained at the same time under the same conditions we detected VAP-1 in normal colon in vessels associated with pericytes and smooth muscle cells as described before. This is consistent with the stated conclusion of the Human Protein Atlas that there is an absence of VAP-1 staining in CRC [16].

Increased or strong VAP-1 expression has been detected on some cancers including head and neck and liver cancers where it has been proposed that VAP-1 supports the recruitment of lymphocytes [7]. In a melanoma mouse model, tumour vessels expressed VAP-1 and VAP-1 knock-out mice demonstrated an impaired ability to form new tumour vessels [9]. In this model, inhibition of VAP-1 reduced tumour-infiltration by CD8+ T cells and MDSCs leading to the conclusion that VAP-1 can support tumour progression via the recruitment of immunosuppressive cells. However, in humans and in keeping with our findings, a reduction of VAP-1 expression by immunohistochemistry was found on vessels in human melanoma tissue compared to peritumoral vessels [23]. In contrast to melanoma tissue, increased VAP-1 immunoreactivity has recently been demonstrated in microvessels in human conjunctival tumours [24] and in human breast cancer, tumour VAP-1 mRNA expression is associated with oestrogen receptor expression and improved prognosis [25, 26]. In contrast, amine oxidase activity was lower in malignant compared to benign tumours in a rat breast cancer model [27]. Thus the role of VAP-1 in tumour progression is likely to depend both on the host and tumour type.

## Conclusions

Our data suggest that VAP-1 is downregulated in human CRC because we found reduced circulating protein levels, lack of detectable mRNA and VAP-1 protein in tumours. Furthermore we were unable to detect VAP-1 in primary tumour endothelial cells isolated from human CRC. The molecular basis of this down regulation is not yet known but it may be part of a mechanism activated by the tumour to prevent the recruitment of anti-tumour immune cells. Given that sVAP-1 is predominantly produced by the hepatic vasculature it was surprising that sVAP-1 levels were reduced in CRC and this suggests the cancer may also have systemic effects in suppressing VAP-1 expression. Whether these low levels of sVAP-1 will prove to have any use as markers of prognosis remains to be seen but the level of overlap between the CRC patients and matched patients would make this unlikely.

## Abbreviations

AOC3: amine oxidase, copper containing-3
CRC: colorectal cancer
GUSB: glucuronidase, beta
IPO8: importin-8
MDSC: myeloid-derived suppressor cells
NC: normal colon
NHS: National Health Service
sVAP-1: soluble VAP-1
